# Improvement of K562 Cell Line Transduction by FBS Mediated Attachment to the Cell Culture Plate

**DOI:** 10.1155/2019/9540702

**Published:** 2019-03-27

**Authors:** Maryam Abbasalipour, Mohammad Ali Khosravi, Sirous Zeinali, Hossein Khanahmad, Morteza Karimipoor, Kayhan Azadmanesh

**Affiliations:** ^1^Department of Molecular Medicine, Biotechnology Research Center, Pasteur Institute of Iran, Tehran 1316943551, Iran; ^2^Department of Molecular Biology and Genetics, Isfahan University of Medical Science, Hezar-Jerib Ave., Isfahan 8174673461, Iran; ^3^Department of Virology, Pasteur Institute of Iran, Tehran 1316943551, Iran

## Abstract

Lentiviral vectors have been used for gene therapy in the clinical phase in recent years. These vectors provide a tool for gene insertion, deletion, or modification in organisms. The K562 human cell line has been used extensively in hematopoietic research. Despite its broad application, it is hard-to-transfection and transduction. So, this study presents a simple method to increase the transduction efficiency of K562 cells with a low multiplicity of infection (MOI) of the virus particle. For this purpose, 24-well plate was coated by 300 *μ*l fetal bovine serum (FBS) before seeding. Then 2×10^4^ K562 cells were seeded in each FBS coated plate. After 24h, K562 cells were attached and doubled. Different amount of lentivirus-based GFP vector according to MOI (5, 10, 15, and 20) along with 8 *μ*g polybrene was added to the attached K562 cells and after 6h cells and viral particle complex were spinfected. Then cells were returned to the plate and incubated in 37°C overnight. After 48h transduction efficiency was established by measuring the GFP-expressing cells by flow cytometry. Flow cytometry analysis showed that, after plate treatment by FBS, 64.5% transduction rate in K562 cells was achieved at MOI=20. Therefore, this method can be an effective and simple way to increase the lentiviral transduction rate for suspended cells such as K562.

## 1. Introduction

The myeloid-erythroid-leukemic K562 cell line is isolated from a patient with chronic myelogenous leukemia in blast crisis. It produces fetal and embryonic hemoglobins and has been used as a model for a broad variety of investigations including the study of human erythroid differentiation, the biology of factors regulating the expression of the embryonic and adult globin genes, and immunotherapy of leukemia. Moreover, K562 cells have been used for the study of differentiation of hematopoietic cells [1–5]. However, cell lines derived from hematological malignancies such as leukemia are poorly transduced with lentiviral particles because these cells are incompatible for a viral method, because certain cognate molecule expression is required on the cell surface for the viral transduction [6]. The previous study has shown that hematopoietic malignancies cell lines such as HL-60 and K562 are semiadherent cells and attached to the positively charged (cationic) cell culture flask surface. In the presence of fibronectin, extracellular matrix molecule, via *α*4*β*1 and *α*5*β*1 integrin molecules which are the surface receptor [6–9]. Fibronectin is a 45 Kd glycoprotein, existing in a soluble form in plasma and serum of all vertebrate, and as an insoluble form, it is located on the normal cell surface and extracellular matrix [10]. This protein is present in fetal bovine serum. Recent studies have shown the soluble fibronectin intercede adhesion and spreading of the cell as used to coat collagen or plastic surfaces. Serum proteins like fibronectin and vitronectin can attach to the cell plate surface and act as a ligand for the specific receptor which is located on the surface of the cell [9–13].

The safety and efficacy of gene delivery strategies as well as transgene expression at a therapeutic level are the required parameters for successful gene therapy.

To achieve successful gene therapy, lentiviral vectors have been developed as an appealing and promising tool because of their potency to integrate into the genome with high efficiency, both dividing and nondividing cells, and permanent expression of transgenes [14–17]. They have been recently used in multiple preclinical and clinical gene therapy trial (e.g., *β*-thalassemia and severe-combined-immunodeficiency disorders) [15].

Since suspended cell lines, mainly leukemia cells, have been used in a wide range of studies, usually low rate of transfection and transduction efficiency cause difficulty of work with these cells. Studies have shown that the electrostatic repulsion between the negatively charged cell and virus envelope causes reduction in transduction rate. To address this concern, some methods including spinoculation, using multiple rounds of transduction, recombinant fibronectin, and adding polycationic reagent were exploited to concentrate the viral particles on the cell surface [18].

Therefore researchers are still trying to establish an easy and effective method for their transfection and transduction. In this regard, we aimed to develop a simple and cost-effective method to enhance the lentiviral transduction efficiency in K562 cells by promotion of cell adhesion to the plate surface through fetal bovine serum to facilitate virus entrance.

## 2. Material and Methods

### 2.1. Lentiviral Vector Production

For generating the lentivirus, 8×10^6^ Lenti-X 293T cells (Clontech) were seeded into 10 cm^2^ plate 24 h prior to transfection in Dulbecco's Modified Eagle Medium (DMEM; Gibco) with 10% FBS, 100 units/ml penicillin, and 100 *μ*g/ml streptomycin at 37°C in a humidified 5% CO_2_ incubator. Third generation lentivirus helper plasmids (pLp1 packaging plasmid, pLp2 regulatory plasmid, and pVSV-G envelope plasmid) and pLOX-CW GFP; Dest plasmid were cotransfected in Lenti-X 293T cell line to produce lentiviral vector particle using lipofectamine 3000 (ThermoFisher) according to manufacturer's instructions. At 6 h posttransfection, the medium was replaced with fresh medium. After 24 h, medium containing viral vector was collected in conical tubes and collection was performed every 6 h until 4 days and stored at 4°C.

### 2.2. Evaluation of Transfection Efficiency

After 48 h, transfection efficiency in Lenti-X 293T cell line was measured by flow cytometry. GFP expression was determined as reporter gene of transfection. The percentage of transfected cells in a cell population.

### 2.3. Lentiviral Vector Concentration

Viral vector supernatant was filtered through cellulose acetate (0.45 *μ*m). Virus concentration was done in high-speed centrifuge (Sigma 3-30 k) 48,000 × g for 2 h. After centrifugation, the supernatant was decanted and viral pellet was resuspended in 200-300 *μ*L cell culture medium, aliquoted in 50 *μ*l, and stored at -80°C [19].

### 2.4. Lentiviral Vector Titration by Flow Cytometry

Following concentration by high-speed centrifuge, serial dilution of the concentrated virus was used to infect HEK 293 cells according to Trono Lab protocol [20] to determine lentiviral vector titer. GFP positive cells were observed using a green fluorescent microscope. The analysis was performed by flow cytometry, using a Cyflow SL (Partec, Germany) and the FlowJo software. The value of GFP expression in cells is an indicator of the entrance of viral vector particles in the cells and then integration and transgene (GFP) expression.

The titer was calculated based on the following formula [20]:

(1) $$\begin{array}{c} Titer\left(\;transducing\;\;units/ml\;\right)=\frac{\text{number of target cells}\left(count\;\;at\;\;day\;\;1\right)\times \left[\left(\%\text{ of GFP}-\text{positive cells}\right)/100\right]}{\text{volume of vector}\left(ml\right)} \end{array}$$

The concentrated lentiviral vector particles were used to transduce K562 cells in a 24-well plate.

### 2.5. K562 Cell Culture

Semiattached K562 (National Cell Bank of Iran, C122) cells were cultured in RPMI 1640 (Gibco) supplemented with 10% FBS (Gibco), penicillin 100 units/ml, and streptomycin 100 *μ*g/ml at 37°C in a humidified 5% CO_2_ incubator overnight.

### 2.6. Fetal Bovine Serum Exposure

Before FBS exposure, 24-well treated cell culture plates (SPL, Korea) were coated by 0.2% glutaraldehyde for 15 min to activate hydroxyl group in a treated surface plate then rinsed twice with ddH_2_O. The plates were coated with FBS (heat-inactivated at 56°C for 30 min) in 300 *μ*l and then incubated for 48 h. Second 24-well plate is used as uncoated (control) plate [9].

### 2.7. K562 Cells Transduction

Before transduction, the FBS coated plates were washed with ddH_2_O twice and then exposed to UV light for 45 min. K562 cells were seeded at a density of 2×10^4^ cells per well (in coated and uncoated plates), incubated at 37°*C* in a humidified 5% CO_2_ incubator overnight. Cell culture medium in plates was removed and about 100 *μ*l medium remained in each well, and 30 min before transduction, the virus was thawed on ice and 8 *μ*g polybrene (Biosettia) was added to 100 *μ*l of the virus in the tube. Viral vector particle-polybrene complex was added to each well, in coated and uncoated plates, in different multiplicities of infection MOI (5, 10, 15, and 20). Plates were shaken gently and placed back in the incubator and incubated at 37°C and 5% C02. Six hours after transduction, K562 cells spinfection was carried out at 800 × g for 70 min at 32°C. After that, the cells were returned to the plates and incubated overnight.

### 2.8. Flow Cytometry Analysis of Transduction Efficiency

Transduction efficiency was evaluated by measuring the percentage of GFP-expressing cells by flow cytometry, using a Cyflow SL (Partec, Germany) and analyzed by FlowJo software. Cells were washed with PBS and then fixed with 1% paraformaldehyde before the analysis.

### 2.9. Data Analysis

Data were analyzed with Student's t-test using GraphPad Prism 6 program. The P-value for statistical significance is defined as P < 0.05. Figure was generated using the GraphPad Prism 6 program.

## 3. Results

### 3.1. Lentiviral Vector Particle Production and Titration

Flow cytometry analysis showed that more than 90% of the Lenti-X 293T cells expressed GFP. So transfection efficiency was measured at ≥90%. The supernatant of cells containing lentiviral vector was concentrated by high-speed centrifuge. Titration by FACS method showed that, after concentration, 2×10^7^ TU/ml on HEK 293 cells was achieved.

### 3.2. FBS Coating and K562 Transduction

To determine the effect of FBS coating on the transduction efficiency, we examined GFP expression of K562 cells cultured in FBS coated and uncoated plates.

**Figure 1:**
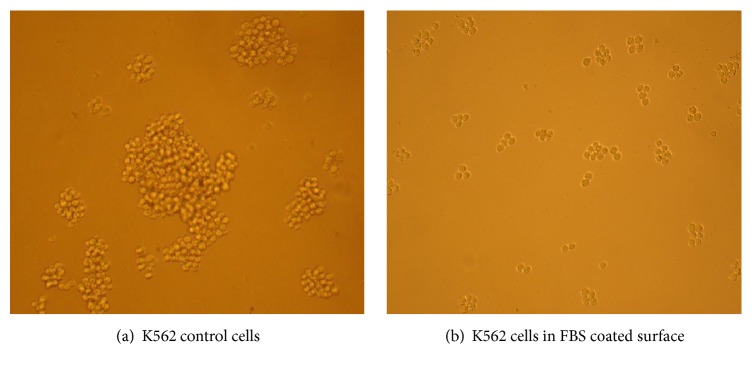
K562 cells cultured in uncoated (control) well and FBS coated plate. (a) K562 control cells in the uncoated plate are in native shape and they are suspended and grow in a clumping form, but in FBS coated surface (b) cells grow in sporadic form and attached to the plate surface. Cells were monitored with Olympus microscope (10X objective).

K562 cells were cultured in FBS coated and uncoated (control) plates before transduction. After 24 h almost all K562 cells in a coated plate with FBS were attached and doubled in number. In an uncoated plate, a few cells were attached and most of them were in the suspension (Figure 1).

**Figure 2:**
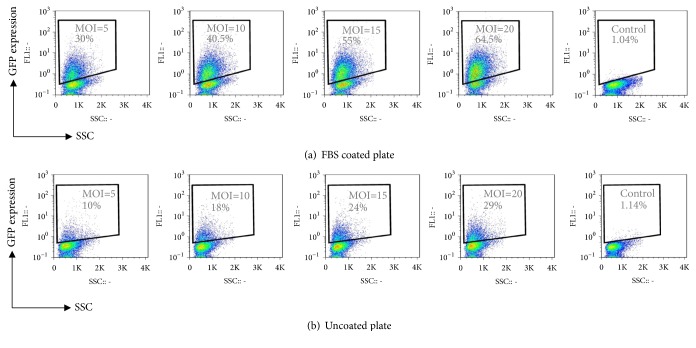
Flow cytometry analysis of GFP gene transfer efficiency and expression in K562 cells transduced by increasing MOI of lentiviral vector particles after 48 h. (a) shows K562 cells which are cultured in FBS coated plate and percentage of GFP expression. (b) shows K562 cells which are cultured in the uncoated plate and percentage of GFP expression. Vertical axes present fluorescent emission (FL-1) and horizontal axes present side scatter (SSC). Values within the gated area show the percentage of GFP gene expression in gated cells. The gate in control nontransduced samples was set to 1 %.

Then the viral vector particles were added to cells in different MOI (5, 10, 15, and 20). After 48h, GFP expression was measured in FBS coated and uncoated groups and the results are shown in Figures 2(a) and 2(b). Flow cytometry analysis showed that, during lentivirus transduction process in both groups, the higher MOI resulted in the more numbers of K562 cells transduced. In uncoated and coated plates, MOI=5 had the lowest rates of transduction (10% and 30%, respectively), while MOI=20 had the highest rate (29% and 64.5%, respectively).

**Figure 3:**
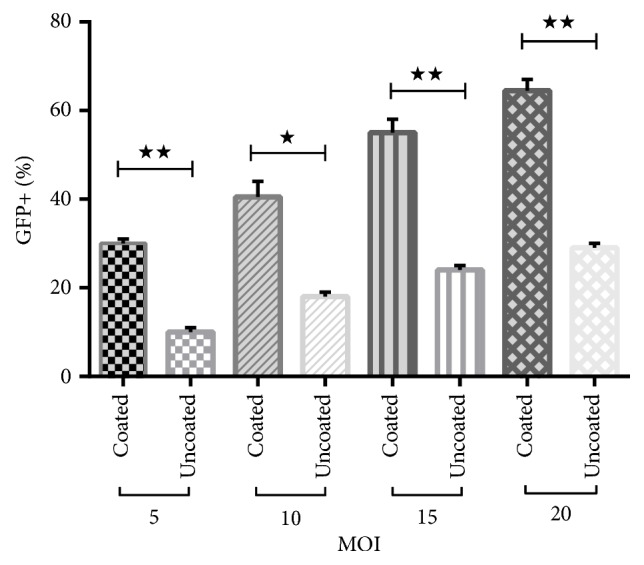
K562 cells transduction efficiency for different MOIs grown in FBS coated plate and uncoated plate (bar: mean ± SEM). To examine the effect of coating FBS on lentiviral infection, K562 was cultured in FBS coating plate which promote cells attachment to plate surface and compared with K562 cells cultured in uncoated plate cells. K562 cells which are grown in coated plate showed significant increase in the transduction percentage compared with cells in uncoated plate group. Asterisk indicates significant difference between two groups by t-test (P < 0.05).*∗*P<0.05; *∗∗*P<0.01.

The transduction efficiencies among different MOIs between two groups (FBS coated and uncoated) were significant: MOI 5; 10 ± 1% versus 30 ± 1, MOI 10; 18 ± 1% versus 40.5 ± 3%, MOI 15; 24 ± 1% versus 55 ± 3% and MOI 20; 29 ± 1% versus 64.5 ± 2.5% (mean ± SEM), respectively (Figures 2 and 3).

## 4. Discussion

Many different gene transfer procedures, such as physical, chemical, and biological methods, have been described over the years to increase the transfection efficiency. Each method has its advantages and disadvantages, so the choice of method depends mostly on the cell type and the purpose of the experiment.

Nowadays lentiviral vectors have been increasingly used for gene therapy application in clinical phase. High gene transfer efficiency and stable integration in the host genome of dividing and nondividing cells are considered as their main advantages, especially when long-term expression is required [21–25].

Although nonviral delivery methods are being developed rapidly, plasmid delivery into hard-to-transfect cells, such as leukemia/lymphoma and stem cells, remains a challenge [21].

Stem cells are a promising therapeutic target for gene therapy, but due to their low gene transfer efficiency by viral vector, stem cell genetic modification has limited success. Several factors such as the activity of the promoter, vector size, viral backbone, packaging cell line, low concentration of viral stocks, inhibitor present in viral stock, transduction conditions and polycationic agents, cell susceptibility to the virus, and virus attachment to the host cells could affect transduction efficiency [26, 27]. For the successful entry of enveloped viruses into the target cells, the specific binding of the cell surface receptors to glycoprotein spikes on the viral membrane surface is also required [28].

Lymphoid cells such as Jurkat T cell, Raji, B cell, and k562 cell lines normally grow in the suspension state. Suspension cell lines are usually hard-to-transfect. The reason for this is that the attachment of transfection complex to the surface of the target cell in suspension culture is reduced [29].

Several methods of gene delivery into the K562 cell have been tested until now. However, their results are not consistent.

Although lipofection protocol seems to be a promising approach for the delivery of large DNA to K562 cells, Hamid et al. [30] showed that K562 cells transfection rate by lipofectamine™ LTX reagent was low (8%). We also ourselves tested K562 cells transfection by lipofectamine 2000, and transfection efficiency result was low (data not shown).

Another method which has been used for K562 cell lines is electroporation. This method yields higher transfection efficiency, but in this method the cell viability is low. In the literature, 40-60% efficiency with 40-80% cell viability has been reported by electroporation of K562 cell line [31, 32].

However, electroporation results are not reproducible, since the condition of the electric pulse is not declared by the company and must be determined empirically for each cell line [31]. We have not been able to exceed 50% transfection rate using ® Cell Line Nucleofector® Kit V (data not shown).

Previous studies have highlighted and focused on the use of this vector in infection of the target cells. Viral vectors can overcome the barriers of gene delivery in hard-to-transfect cells. Usually in these cell types, for achieving higher transduction efficiency by a lentiviral vector, high MOI is required. However, more viral vector particles can be toxic to cells.

Previously published experiments have shown that K562 transduction efficiency is not the same in all of them and for high transduction rate, high MOI is needed or a new method for transduction should be developed. Previous studies have employed a high-throughput screen to identify the polycationic reagents, small molecules, and cytokines effect on lentiviral vector transduction but just a small number of them are shown to improve the transduction efficiency [18, 33].

Schakowski and et al. [34] analyzed various gene transfer methods to transfect K562 cell line. In this way, their study showed that, for adenoviral infection, high MOI=200 of adenovirus particle is necessary to transduce 49% K562 cells, electroporation and particle bombardment (Gene gun) were not effective, and transfection rate was 15.5% and 1.5%, respectively. They established new nucleofection method with 75% transfection rate and 34.0 ± 14.6% dead cells.

Prior studies have shown that promotion of cells attachment to the plate surface in the presence of polycationic reagents, to reduce electrostatic interference between virus and cell surface, or adhesion of cells onto the cationic surface in the presence of fibronectin presumably facilitates more efficient virus contact between target cells and viral vector particles and improved transduction [11, 12, 35]. In this study, we tested the attachment and transduction efficiency of semiattached K562 cells which were grown in FBS coated plates. We have shown that, in the presence of coated FBS, after 24 h K562 cells are attached to the plate surface. This result agrees with the previous studies [9].

The transduction efficiency tends to increase as the MOI increases in FBS coated plate. We achieved transduction efficiency for K562 cells up to 64.5% at MOI=20.

## 5. Conclusion

Our data demonstrate that fetal bovine serum provides a suitable, simple, reproducible, and inexpensive method to enhance the transduction efficiency of suspension cell lines by lentiviral vectors in low MOI. It is important to note that researchers choose their transduction method based on cell type. One method may be suboptimal for some cell types but may work well for a particular cell type. Therefore, the method described in this study may not work for all type of cells.

## Data Availability

The data used to support the findings of this study are available from the corresponding author upon request.
